# Glioma Type Prediction with Dynamic Contrast-Enhanced MR Imaging and Diffusion Kurtosis Imaging—A Standardized Multicenter Study

**DOI:** 10.3390/cancers16152644

**Published:** 2024-07-25

**Authors:** Leonie Zerweck, Till-Karsten Hauser, Uwe Klose, Tong Han, Thomas Nägele, Mi Shen, Georg Gohla, Arne Estler, Chuanmiao Xie, Hongjie Hu, Songlin Yang, Zhijian Cao, Gunter Erb, Ulrike Ernemann, Vivien Richter

**Affiliations:** 1 Department of Diagnostic and Interventional Neuroradiology, University Hospital Tuebingen, 72076 Tuebingen, Germany; till-karsten.hauser@med.uni-tuebingen.de (T.-K.H.); uwe.klose@med.uni-tuebingen.de (U.K.); vivien.richter@med.uni-tuebingen.de (V.R.); 2 Tianjin Huanhu Hospital, Tianjin 300350, China; 3 Department of Radiology, Beijing Tian Tan Hospital, Capital Medical University, Beijing 100050, China; 4 Department of Medical Imaging, Sun Yat-sen University Cancer Center, Guangzhou 510060, China; 5 Department of Radiology, Sir Run Run Shaw Hospital, School of Medicine, Zhejiang University, Hangzhou 310018, China; 6 Department of Radiology, The Fifth Affiliated Hospital of Sun Yat-sen University, Zhuhai 519082, China; 7 Department of Radiology, The First Affiliated Hospital of Zhejiang Chinese Medical University, Hangzhou 310053, China; 8 Bracco Group, Medical and Regulatory Affairs, 78467 Konstanz, Germany

**Keywords:** brain tumor, glioma, dynamic contrast-enhanced magnetic resonance imaging, diffusion kurtosis imaging

## Abstract

**Simple Summary:**

The identification of gliomas and the differentiation between different types is essential to evaluate patients’ prognosis and guide optimal clinical management. The ideal multiparametric magnetic resonance imaging (MRI) protocol for the assessment of gliomas is a current topic of research. This study aimed to explore the performance of dynamic contrast-enhanced (DCE) MRI and diffusion kurtosis imaging (DKI) in differentiating molecular subtypes of adult-type diffuse gliomas. The results showed that a combined evaluation of DCE-MRI and DKI parameters reveals the best prediction of high-grade vs. low-grade gliomas, IDH1/2 wildtype vs. mutated gliomas, and astrocytomas/glioblastomas vs. oligodendrogliomas.

**Abstract:**

The aim was to explore the performance of dynamic contrast-enhanced (DCE) MRI and diffusion kurtosis imaging (DKI) in differentiating the molecular subtypes of adult-type gliomas. A multicenter MRI study with standardized imaging protocols, including DCE-MRI and DKI data of 81 patients with WHO grade 2–4 gliomas, was performed at six centers. The DCE-MRI and DKI parameter values were quantitatively evaluated in ROIs in tumor tissue and contralateral normal-appearing white matter. Binary logistic regression analyses were performed to differentiate between high-grade (HGG) vs. low-grade gliomas (LGG), IDH1/2 wildtype vs. mutated gliomas, and high-grade astrocytic tumors vs. high-grade oligodendrogliomas. Receiver operating characteristic (ROC) curves were generated for each parameter and for the regression models to determine the area under the curve (AUC), sensitivity, and specificity. Significant differences between tumor groups were found in the DCE-MRI and DKI parameters. A combination of DCE-MRI and DKI parameters revealed the best prediction of HGG vs. LGG (AUC = 0.954 (0.900–1.000)), IDH1/2 wildtype vs. mutated gliomas (AUC = 0.802 (0.702–0.903)), and astrocytomas/glioblastomas vs. oligodendrogliomas (AUC = 0.806 (0.700–0.912)) with the lowest Akaike information criterion. The combination of DCE-MRI and DKI seems helpful in predicting glioma types according to the 2021 World Health Organization’s (WHO) classification.

## 1. Introduction

The identification of gliomas and the differentiation between types, according to the 2021 World Health Organization (WHO) classification of tumors of the central nervous system (CNS), is essential to evaluate patients’ prognosis and guide optimal clinical management [1,2,3]. The definitive grading relies on histopathological and molecular examinations of stereotactic biopsy or resection [4,5,6,7]. However, a reliable non-invasive tumor assessment is required for follow-up of suspected low-grade gliomas (LGG) when patients are not eligible for surgery or for the monitoring of potential tumor recurrence [8].

The ideal multiparametric magnet resonance imaging (MRI) protocol in the neuroradiological assessment of gliomas is an ongoing topic of research [9].

Perfusion-weighted imaging (PWI) is commonly used to evaluate gliomas [10,11,12,13]. The most frequently used MR perfusion technique in clinical practice is dynamic susceptibility contrast-enhanced (DSC) MRI [10,14]. However, dynamic contrast-enhanced (DCE) MRI provides more reliable quantification of the blood–brain barrier and microvasculature permeability [10,14] and a higher spatial resolution than DSC MRI [10]. DCE-MRI is supposed to provide high diagnostic accuracy in differentiating between LGG and high-grade gliomas (HGG) [10,15].

Diffusion-weighted imaging (DWI) is also routinely used in oncological neuroimaging [7,12,13,16]. A standard DWI assumes that the displacement of water molecules follows a Gaussian distribution [6,16,17]. However, complex biological tissue with cell membranes that generate water compartments leads to a non-Gaussian behavior of water diffusion [6,16,17]. Diffusion kurtosis imaging (DKI) is an advanced diffusion imaging technique that does not assume a Gaussian distribution of water molecules and quantifies the degree of deviation from a Gaussian behavior [6,16,18,19]. DKI provides additional microstructural information, allowing for a better glioma grading than conventional diffusion parameters and morphological imaging [18,20,21].

This study aimed to quantitatively compare the diagnostic efficiency of the DCE-MRI and DKI parameters and to investigate whether a combined multiparametric tumor assessment based on DCE-MRI and DKI can improve the diagnostic quality of tumor grading and molecular subtype identification.

## 2. Materials and Methods

### 2.1. Study Design

A prospective multicenter study at six neurosurgical centers in China was performed. The study was performed in accordance with the principles of the Declaration of Helsinki and approved by the local ethics committee at each center. Written informed consent was obtained from all participants.

### 2.2. Patients

One hundred and eight patients with suspected cerebral glioma were enrolled and imaged with a standardized MR imaging protocol. The inclusion criteria were a suspected supratentorial cerebral glioma and a scheduled cerebral tumor biopsy and/or surgery, including a histopathological evaluation within four weeks following the date of the study’s MRI. The exclusion criteria were MRI contraindications (e.g., pace-maker, metal implants, pregnancy, contrast agent allergy, severe renal impairment defined as a glomerular filtration rate (GFR)/estimated GFR (eGFR) < 30 mL/min, severe claustrophobia, etc.) and radiotherapy or chemotherapy before the biopsy/surgery.

### 2.3. MR Imaging

All patients were examined on a 3T MRI scanner, all of which were Siemens-manufactured equipment, to increase the comparability of the acquired images at all sites.

The standardized imaging protocol included a conventional MRI (axial T1 spin-echo (SE)/fast spin-echo (FSE) sequence pre- and post-contrast, axial T2 FLAIR, 3D-T1 gradient-echo (GRE) sequence post-contrast), and additionally DCE-MRI and DWI.

The DCE-MRI was acquired with a dynamic 3D-T1 volumetric interpolated breath-hold examination (VIBE) sequence (TR 4 ms, TE 1.8 ms, voxel size 1.5 × 1.1 × 4.0 mm, number of excitations (NEX) 1, and parallel acquisition technique (PAT) 2). Three flip angles (FA) (6°, 9°, 15°) were used for the T1 quantification. After administration of Gadobenate dimeglumine (MultiHance; Bracco) at a single dose of 0.1 mmol/kg and an injection rate of 4 mL/s, dynamic T1 measurements were performed.

DWI was performed using a 2D echo-planar imaging (EPI) sequence with multiple b values of 0, 500, 1000, 1500, 2000, and 2500 s/mm^2^ for diffusion kurtosis analysis (TR 5900 ms, TE 95 ms, voxel size 2.0 × 2.0 × 5.0 mm, NEX 2, PAT 2, number of diffusion-encoding directions 6/b value, and EPI factor 128).

### 2.4. Image Analysis

MRI data post-processing and analyses were performed off-site.

The values of the following DCE-MRI and DWI parameters were calculated using scripts written in MATLAB (R2018b (The MathWorks, Inc., Natick, MA, USA; http://www.mathworks.com)) accessed on 2 June 2024: Apparent diffusion coefficient (ADC), mean kurtosis (MK), Ktrans, Kep, vp, ve, cerebral blood volume (CBV), time to peak (TTP), peak, area under the curve (AUC_DCE_), wash in, and wash out. The ADC was derived from the DKI model and not from a monoexponential model. The full diffusion signal S is described as

ln(S/S_0_) = −bADC + (bADC)^2^ K/6 (1)

where K is the kurtosis. Multiple directions were used to improve the signal-to-noise ratio and for the independence of accidental local directional influences on the diffusion measurement.

**Figure 1:**
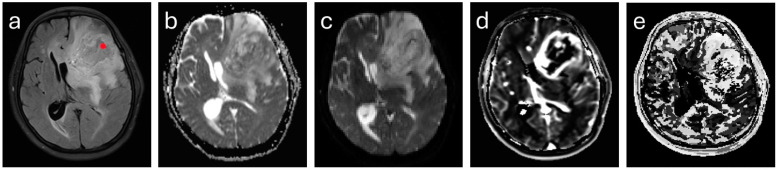
Exemplary maps of one patient with glioblastoma in the left frontal lobe. FLAIR (**a**), apparent diffusion coefficient (ADC) (**b**), mean kurtosis (MK) (**c**), dynamic contrast-enhanced (DCE) MRI parameter Ktrans (**d**), and time to peak (TTP) (**e**). The red mark indicates the biopsy ROI.

After surgery, the performing neurosurgeon drew a small circular region of interest (ROI) on the FLAIR maps in the region where the biopsy was taken. The biopsy sites were determined by the performing neurosurgeon based on clinical criteria such as good surgical accessibility, avoiding important functional regions (e.g., the central region), and a high probability of high-grade malignancy on MR imaging (e.g., strong contrast enhancement). Small ROIs were defined (radius 5 pixels = 2.2 mm) in order to minimize the possibility of including healthy tissue; thus, the ROI did not always cover the entire biopsy region. In each patient, one biopsy ROI was evaluated and included in the study (see Supplementary Figure S1), and another control ROI was drawn in the contralateral normal-appearing white matter (NAWM). The positions and sizes of the ROIs were transferred to the parameter maps by co-registration of the maps and the mean ROI values of each parameter were calculated. Exemplary maps can be seen in Figure 1.

### 2.5. Postoperative Tumor Grading

The postoperative pathology reports were analyzed by a pathologist for histopathological diagnosis, tumor grade, and molecular tumor markers according to the 2021 WHO classification of brain tumors [5]. LGG were defined as WHO grade 2 gliomas, and HGG as WHO grades 3 and 4 gliomas.

### 2.6. Statistical Analysis

All statistical analyses were performed using SPSS Statistics (IBM Corp. Released 2021. IBM SPSS1 Statistics for Windows, version 28.0. Armonk, NY, USA: IBM Corp.).

First, the mean ROI values of the biopsy region were investigated for group differences between (i) low-grade astrocytomas (WHO grade 2) vs. high-grade astrocytomas (IDH1/2 mutated) (WHO grades 3 and 4) and glioblastomas (IDH1/2 wildtype) (WHO grade 4), and (ii) IDH1/2 wildtype vs. IDH1/2 mutated adult-type gliomas and (iii) oligodendrogliomas (IDH1/2 mutated 1p/19q codeletion) (WHO grade 3) vs. glioblastomas (IDH1/2 wildtype) (WHO grade 4) and astrocytomas (IDH1/2 mutated) (WHO grade 4 and 4).

For this purpose, the Shapiro–Wilk test for normal distributions was applied for all DKI and DCE-MRI parameters. The Levené test was performed to test for homogeneous variance. Next, either an unpaired *t*-test (in case of homogeneous variance)/or a Mann–Whitney U test (in case of inhomogeneous variance and non-normal distribution)/Welch test (in case of inhomogeneous variance and normal distribution) was performed to test for differences between the above-mentioned groups. Tests of the 12 a priori hypotheses (differences in ADC, MK, Ktrans, Kep, vp, ve, CBV, TTP, Peak, AUC_DCE_, wash in, and wash out) were conducted using Bonferroni-adjusted alpha levels of 0.004 per test (0.05/12). Group comparisons were also performed in the NAWM to verify the results.

Second, the ability of all parameters and models with combined DCE-MRI and DKI parameters to discriminate between (i) LGG vs. HGG, (ii) IDH1/2 wildtype vs. IDH1/2 mutated gliomas, and (iii) high-grade oligodendroglial vs. high-grade astrocytic gliomas (see more detailed descriptions of the groups above) was evaluated.

For this purpose, univariate binary logistic regression analyses were performed to assess the diagnostic values of all the DCE-MRI and DKI parameters to differentiate between (i) LGG vs. HGG, (ii) IDH1/2 wildtype vs. IDH1/2 mutated gliomas, and (iii) high-grade oligodendroglial vs. high-grade astrocytic gliomas. Afterward, forward binary logistic regression analyses were performed with the distinction between (i) LGG vs. HGG, (ii) IDH1/2 wildtype vs. IDH1/2 mutated gliomas, and (iii) high-grade oligodendroglial vs. high-grade astrocytic gliomas as dependent variables and the DCE-MRI and DKI parameters as the independent variables. The Akaike information criterion (AIC) was calculated to account for the number of predictors.

Receiver operating characteristic (ROC) curves were generated for each single parameter and for the calculated regression models with the lowest AIC to determine the area under the curve (AUC), sensitivity, and specificity. For each ROC curve analysis, the result with the highest Youden index was defined as the optimal cut-off value.

## 3. Results

### 3.1. Patients

In total, 108 patients with the tentative diagnosis of cerebral glioma were enrolled. Three patients underwent biopsy only, and the others underwent surgical resection. After a review of histopathology, 91 patients with supratentorial gliomas of WHO grades 2–4 were included in the further analysis. Data sets of 10 patients were excluded due to insufficient image quality. Finally, the data sets of 81 patients were included. In seven patients, the IDH mutation status was missing, but their data sets were nonetheless included due to the detailed histopathologic reports with other molecular characteristics. Patients’ characteristics can be seen in Table 1.

### 3.2. Evaluation of the Individual and Combined DCE-MRI and DKI Parameters

#### 3.2.1. LGG versus HGG

**Figure 2:**
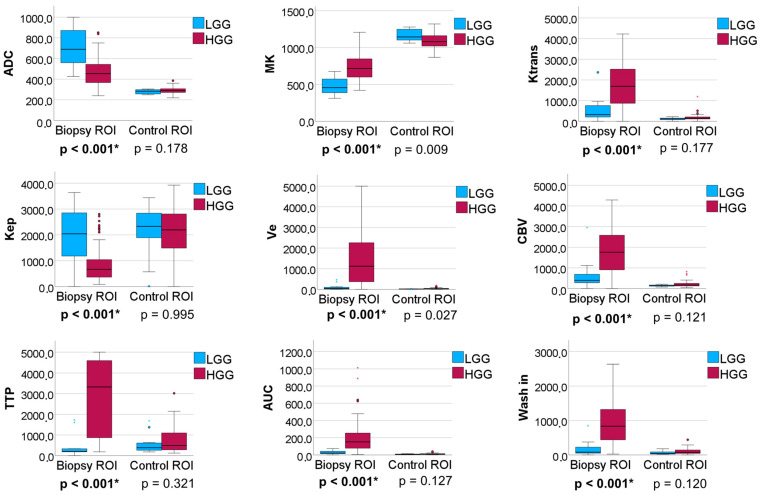
Boxplots illustrating the group comparison of diffusion-weighted imaging (DWI)/diffusion kurtosis imaging (DKI) parameters and dynamic contrast-enhanced (DCE) MRI parameters between patients with high-grade gliomas and low-grade gliomas in biopsy regions of interest (ROI) (left) and contralateral control ROIs (right). Only significant group differences are shown, while all group comparisons can be seen in Supplementary Figure S2. ADC = apparent diffusion coefficient, MK = mean kurtosis, CBV = cerebral blood volume, TTP = time to peak, and AUC = area under the curve. An asterisk (*) marks significant correlation at *p* < 0.004.

Boxplots of all group comparisons between LGG and HGG of all individual parameters are shown in Supplementary Figure S2. Significant differences between the biopsy ROIs of LGG and HGG were found for Ktrans (*p* < 0.001), Kep (*p* < 0.001), Ve (*p* < 0.001), CBV (*p* < 0.001), TTP (*p* < 0.001), AUC_DCE_ (*p* < 0.001), wash in (*p* < 0.001), ADC (*p* < 0.001) and MK (*p* < 0.001) (see Figure 2). In the control ROIs of NAWM, the same group comparisons revealed no significant differences for any of the parameters.

**Figure 3:**
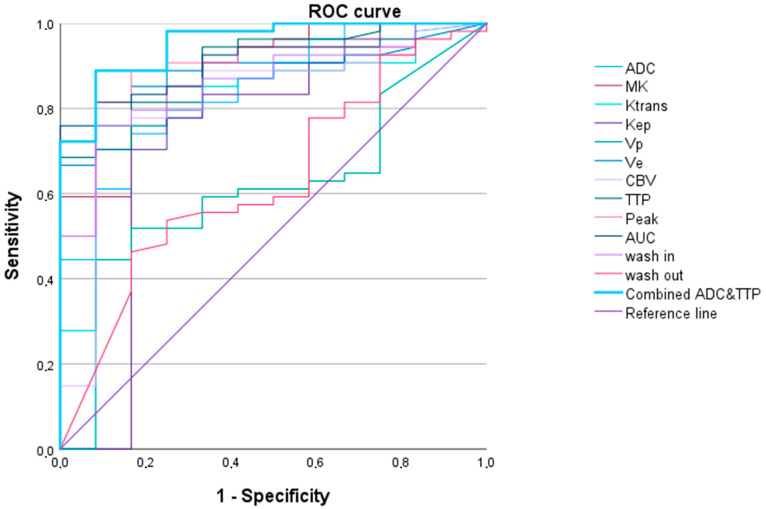
Receiver operating characteristic curves for all parameters in differentiating between high-grade gliomas and low-grade gliomas for apparent diffusion coefficient (ADC), mean kurtosis (MK), and dynamic contrast-enhanced (DCE) MRI parameters (Ktrans, Kep, Vp, Ve, cerebral blood volume (CBV), time to peak (TTP), peak, area under the curve (AUC_DCE_), wash in, and wash out). The single parameter with the highest AUC was MK (AUC = 0.884 (0.788–0.981)). A combined approach including ADC and TTP revealed a higher AUC (954 (0.900–1.000)).

The ROC curve analysis showed high AUC values for the DCE-MRI parameters (up to 0.910 (0.840–0.890) (AUC)) and DWI/DKI parameters (up to 0.884 (0.788–0.981) (MK)) (see Table 2 and Figure 3).

Multivariant binary logistic regression analysis and ROC curve analysis revealed that a combined evaluation of ADC and TTP best predicted LGG vs. HGG with the lowest AIC and highest AUC of 0.954 (0.900–1.000) (see Table 2 and Figure 3).

#### 3.2.2. IDH 1/2 Mutated versus IDH 1/2 Wildtype Gliomas

**Figure 4:**
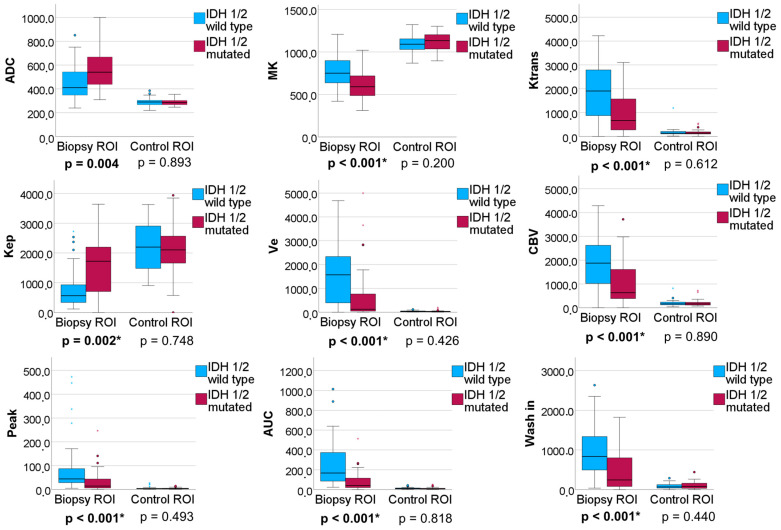
Boxplots illustrating the group comparison of diffusion-weighted imaging (DWI)/diffusion kurtosis imaging (DKI) parameters and dynamic contrast-enhanced (DCE) MRI parameters and neurite orientation dispersion between patients with high-grade gliomas and low-grade gliomas in biopsy regions of interest (ROIs) (left) and contralateral control ROIs (right). Only significant group differences are shown, while all group comparisons can be seen in Supplementary Figure S3. MK = mean kurtosis, CBV = cerebral blood volume, TTP = time to peak, AUC = area under the curve. An asterisk (*) marks significant correlation at *p* < 0.004.

Significant group differences between the biopsy ROIs of IDH 1/2 mutated and IDH 1/2 wildtype gliomas were found for ADC (*p* = 0.004), Ktrans (*p* < 0.001), Kep (*p* = 0.002), Ve (*p* < 0.001), CBV (*p* < 0.001), peak (*p* < 0.001), AUC_DCE_ (*p* < 0.001), wash in (*p* = 0.001), and MK (*p* < 0.001) (see Figure 4 and Supplementary Figure S3). No significant differences were found in the NAWM.

**Figure 5:**
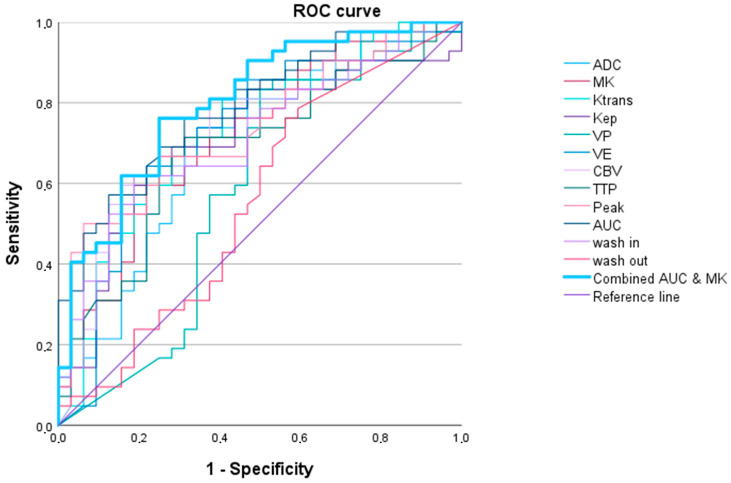
Receiver operating characteristic curves for all parameters in differentiating between IDH1/2 wildtype vs. IDH1/2 mutated gliomas for apparent diffusion coefficient (ADC), mean kurtosis (MK), and dynamic contrast-enhanced (DCE) MRI parameters (Ktrans, Kep, Vp, Ve, cerebral blood volume (CBV), time to peak (TTP), peak, area under the curve (AUC_DCE_), wash in, and wash out). The single parameter with the highest AUC was AUC_DCE_ (AUC = 0.791 (0.609–0.891)). The multimodal approach, including AUC_DCE_ and MK, showed a slightly higher AUC of 0.802 (0.702–0.903).

In the ROC curve analysis, AUC values of the DCE-MRI parameters of up to 0.791 (0.609–0.891) (AUC_DCE_) and AUC values of the DWI/DKI parameters of up to 0.718 (0.601–0.825) (MK)) were detected (see Table 3 and Figure 5).

The regression model with the lowest AIC included the parameters MK and AUC_DCE_. The ROC curve analysis depicted an AUC of 0.802 (0.702–0.903), which was higher than all AUC values of the individual DCE-MRI and DKI (see Table 3 and Figure 5).

#### 3.2.3. High-Grade Oligodendroglial versus High-Grade Astrocytic Gliomas

Boxplots showing the group comparison of all individual parameters are shown in Supplementary Figure S4. The comparison between the DCE-MRI and DKI parameters of oligodendroglial and astrocytic gliomas revealed neither in the biopsy ROIs nor in the NAWM significant group differences.

**Figure 6:**
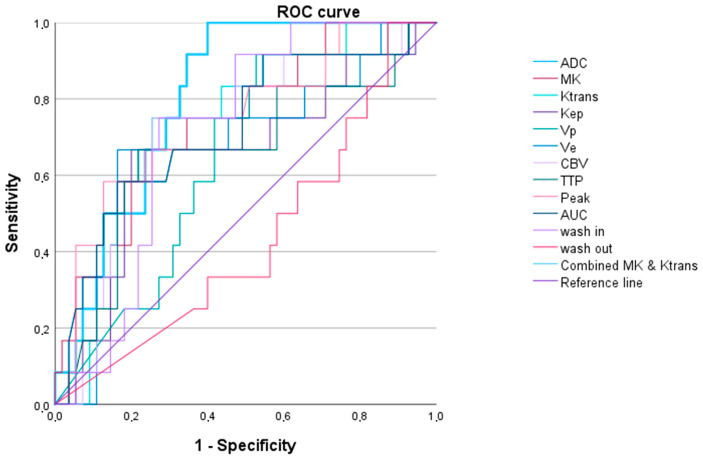
Receiver operating characteristic curves for all parameters in differentiating between high-grade oligodendroglial and astrocytic gliomas for apparent diffusion coefficient (ADC), mean kurtosis (MK), and dynamic contrast-enhanced (DCE) MRI parameters (Ktrans, Kep, Vp, Ve, cerebral blood volume (CBV), time to peak (TTP), peak, area under the curve (AUC_DCE_), wash in, and wash out). The single parameter with the highest AUC was MK (AUC = 0.741 (0.592–0.890)). A combined approach including Ktrans and MK revealed a higher AUC (0.806 (0.700–0.912)).

The ROC curve analysis revealed AUC values of the DCE-MRI parameters of up to 0.739 (0.589–0.880) (Ktrans) and AUC values of the DWI/DKI parameters of up to 0.741 (0.592–0.890) (MK) (see Table 4 and Figure 6).

The multivariant binary logistic regression analysis and ROC curve analysis indicated that a model that included the parameters Ktrans and MK best discriminated high-grade oligodendroglial and astrocytic gliomas with the lowest AIC and highest AUC of 0.806 (0.700–0.912) (see Table 4 and Figure 6).

## 4. Discussion

The aim of this study was to explore the diagnostic performance of combined DKI and DCE-MRI in grading and typing gliomas.

The 2021 updated WHO classification integrates molecular data into the typing, subtyping, and grading of CNS tumors and has a decisive impact on patients’ therapy selection and the development of therapeutic trials [1]. Estimation of tumor grading is no longer the most important aspect of MRI diagnostics, especially for non-increasing LGG [22]. For this reason, we focused not only on the group comparisons between (i) LGG vs. HGG but also investigated the differences between (ii) IDH1/2 wildtype vs. IDH1/2 mutated gliomas and (iii) high-grade oligodendroglial vs. high-grade astrocytic gliomas.

The comparison between LGG vs. HGG showed significant differences for many DCE-MRI and DWI/DKI parameters. As expected, these differences were not observed in the control regions of NAWM. The ROC curve analysis revealed that most of the DCE-MRI and DWI/DKI parameters had significant diagnostic values. However, we showed that the combined evaluation approach of the DWI-derived parameter ADC and the DCE-MRI-derived parameter TTP leads to the best differentiation between LGG vs. HGG with the highest AUC and lowest AIC. The result of this study—that the combined evaluation of DCE-MRI and diffusion imaging parameters results in a higher diagnostic accuracy than the single parameters alone—is in line with the study of Arevalo-Perez et al. [23] and the study by Richter et al., who emphasize the higher diagnostic confidence of a combined approach [24]. The DCE-MRI differences between HGG and LGG might be attributed to the higher vascularity and permeability in HGG than LGG [23], while differences in diffusion imaging could be explained by the higher cellularity expected in HGG than in LGG [23].

IDH is considered the most important marker for the diagnosis and prognosis of gliomas [25]. Significant differences between IDH1/2 wild \type and IDH1/2 mutated gliomas were detectable for many DCE-MRI and DWI/DKI parameters. Earlier studies indicated a correlation between the IDH mutational status and the angiogenesis and cell proliferation in gliomas, serving as an explanation for the observed differences [25,26]. With regard to the IDH1/2 mutational status, our study also showed that a combined evaluation of the DCE-MRI parameter AUC_DCE_ and the DKI parameter MK reveals the best prediction of the IDH1/2 mutational status. This result is in accordance with previous studies that demonstrated higher diagnostic confidence in a combined DCE-MRI/DKI approach than of the individual parameters [24] and reported the highest diagnostic accuracy when combining the texture analysis of DCE-MRI and histogram analysis of the DKI parameters [25].

Oligodendrogliomas are characterized by a better prognosis than astrocytomas/glioblastomas, partly because they are uniquely sensitive to chemotherapy [27,28]. At a Bonferroni-adjusted significance level, we did not find any significant differences in the DCE-MRI and DKI parameters between oligodendroglial and astrocytic gliomas, which is in accordance with the results of Gupta et al., who also failed to discriminate between oligodendroglioma and astrocytoma with DCE-MRI [27]. Nevertheless, it should be noted that Bonferroni correction is one of the most conservative approaches to correct for multiple comparisons [29], and some parameters, e.g., ADC and Ktrans, showed considerable, albeit not significant, differences. Furthermore, our study showed that the combined evaluation of the DCE-MRI parameter Ktrans and the DKI parameter MK leads to a better differentiation between high-grade oligodendroglial and high-grade astrocytic gliomas than all single parameters, which is in line with the study of Richter et al. [24].

This study has some limitations. One limitation was the limited sample size in some subgroups, e.g., oligodendrogliomas, due to the prospective study design, leading to a distribution of included tumor types according to their prevalences. A larger sample size might reveal significant differences between oligodendroglial and astrocytic gliomas.

Furthermore, the majority of the tumors included were WHO grade 4 tumors, which may have biased the results. We performed additional binary logistic regression analyses and ROC curve analyses, in which the three group comparisons were performed, excluding WHO grade 4 tumors (see Supplementary Table S1). The regression analyses showed that the combination of DCE and DWI parameters did not result in a better prediction of the tumor classification than the best-predicting parameters alone. However, these supplementary analyses are not conclusive due to the small number of cases (n = 19; n = 32; n = 20).

Another limitation was that secondary brain tumors were not investigated. In this study, we chose a relatively large slice thickness for the DWI data, which has the disadvantage of a lower spatial resolution. Nevertheless, we selected this slice thickness, as a smaller slice thickness would result in a longer acquisition time. Longer acquisition time must be critically considered in a routine MRI protocol with multiple sequences, especially in patients with brain tumors, as prolonged acquisition time is associated with reduced patient compliance, more motion artifacts, and, thus, poorer image quality.

In recent years, preclinical research on multi-compartment diffusion MRI models (e.g., vascular, extracellular, and restricted diffusion for cytometry in tumor (VERDICT) or imaging microstructural parameters using limited spectrally edited diffusion (IMPULSED)) revealed promising results [30,31,32,33]. Future clinical studies could investigate the additional value of different multi-compartment models in combined multiparametric tumor assessments.

Nevertheless, this is the first prospective multicenter study that quantitatively assesses the tumor grade and type prediction in adult-type gliomas by means of the DCE-MRI and DKI parameters. The ROI placement in tumor tissue, where histopathological sampling was performed, ensured a comparison with the diagnostic gold standard histopathology. An important finding is that a combined evaluation of perfusion and diffusion MRI leads to a better prediction of HGG vs. LGG, IDH1/2 wildtype vs. IDH1/2 mutated gliomas, and high-grade oligodendroglial vs. high-grade astrocytic gliomas, even when taking a different number of predictors into account.

## 5. Conclusions

This multicenter study revealed that the combination of DCE-MRI and diffusion imaging parameters is helpful in predicting glioma types according to the 2021 WHO classification.

## Figures and Tables

**Table 1:** Patient characteristics.

Patients enrolled	108
Patients included	81
Patients excluded due to histopathological diagnosis	17
Patients excluded due to insufficient MRI quality	10
Mean age of the included patients ± SD	45.1 ± 14.8
Female/male ratio	1:1.14
Diffuse Astrocytoma (WHO grade 2)	12 (14.8%)
Anaplastic Astrocytoma (WHO grade 3)	7 (8.6%)
Oligodendroglioma (WHO grade 2)	3 (3.7%)
Oligodendroglioma (WHO grade 3)	12 (14.8%)
Glioblastoma (WHO grade 4)	47 (58.0%)
WHO grade 2	15 (18.5%)
WHO grade 3	19 (23.5%)
WHO grade 4	47 (58.0%)
High-grade glioma (WHO grade 3 and 4)	66 (81.5%)
Low-grade glioma (WHO grade 2)	15 (18.5%)
IDH 1/2 wildtype	32 (39.5%)
IDH 1/2 mutation	42 (51.9%)

**Table 2:** Diagnostic performance of all dynamic contrast-enhanced (DCE) MRI and diffusion-weighted imaging (DWI) parameters and the combined approaches based on the multivariant binary regression analyses in predicting WHO grade 2 vs. WHO grade 3 adult-type gliomas.

	WHO Grade 2 vs. WHO Grades 3 and 4 Adult-Type Gliomas					
	AUC (95% Confidence Interval)	*p*-Value	Cut-Off Value ^1^	Sensitivity	Specificity	AIC
**Ktrans**	0.819 (0.695–0.944)	0.001	1083.0	0.704	0.917	50.101
**Kep**	0.738 (0.538–0.938)	0.010	388.4	0.704	0.833	53.158
**Vp**	0.610 (0.445–0.775)	0.235	1272.1	0.444	0.917	62.937
**Ve**	0.885 (0.803–0.967)	<0.001	133.4	0.852	0.833	40.898
**CBV**	0.821 (0.682–0.960)	0.001	827.9	0.778	0.833	50.917
**TTP**	0.902 (0.822–0.982)	<0.001	1769.4	0.685	1.000	43.158
**Peak**	0.895 (0.804–0.986)	<0.001	11.9	0.889	0.883	41.096
**AUC_DCE_**	0.910 (0.840–0.980)	<0.001	76.2	0.759	1.000	37.911
**wash in**	0.860 (0.761–0.958)	<0.001	13,334.5	0.759	0.917	45.400
**wash out**	0.631 (0.461–0.801)	0.158	4.3	0.463	0.833	60.495
**ADC**	0.844 (0.732–0.956)	<0.001	542.1	0.741	0.833	48.012
**MK**	0.884 (0.788–0.981)	<0.001	551.5	0.852	0.750	41.893
**TTP and ADC ^2^**	**0.954 (0.900–1.000)**	**<0.001**	**0.8**	**0.889**	**0.917**	**28.532**

AUC = area under the curve, AIC = Akaike information criterion, CBV = cerebral blood volume, TTP = time to peak, AUC_DCE_ = area under the curve, ADC = apparent diffusion coefficient, and MK = mean kurtosis. ^1^ Apart from the combined approaches, cut-off values of the raw data are shown, not those of the univariate binary logistic regressions. ^2^ Weights of the logistic regression in parentheses: TTP (0.001) and ADC (−0.01); the parameter/combination with highest AUC and lowest AIC is highlighted in thick letters.

**Table 3:** Diagnostic performance of all dynamic contrast-enhanced (DCE) MRI and diffusion-weighted imaging (DWI) parameters and the combined approaches based on the multivariant binary regression analyses in predicting IDH1/2 wildtype vs. IDH1/2 mutated adult-type gliomas.

	IDH1/2 Wildtype vs. IDH1/2 Mutated Adult-Type Gliomas					
	AUC (95% Confidence Interval)	*p*-Value	Cut-Off Value ^1^	Sensitivity	Specificity	AIC
**Ktrans**	0.731 (0.614–0.849)	0.001	1692.2	0.810	0.594	89.848
**Kep**	0.710 (0.588–0.831)	0.002	842.3	0.667	0.750	91.636
**Vp**	0.583 (0.441–0.724)	0.226	1303.0	0.833	0.500	99.564
**Ve**	0.726 (0.604–0.848)	0.001	366.3	0.643	0,781	94.220
**CBV**	0.738 (0.622–0.854)	<0.001	1734.6	0.810	0.625	91.196
**TTP**	0.685 (0.562–0.807)	0.007	2000.3	0.714	0.688	95.088
**Peak**	0.738 (0.625–0.850)	<0.001	13.0	0.500	0.938	94.056
**AUC_DCE_**	0.791 (0.609–0.891)	<0.001	118.8	0.762	0.688	83.677
**wash in**	0.718 (0.602–0.834)	0.001	439.5	0.619	0.813	92.798
**wash out**	0.555 (0.419–0.692)	0.416	1.0	0.786	0.406	103.058
**ADC**	0.699 (0.576–0.821)	0.004	468.8	0.738	0.656	94.858
**MK**	0.718 (0.601–0.835)	0.001	620.9	0.595	0.781	91.430
**AUC_DCE_ and MK ^2^**	**0.802 (0.702–0.903)**	**<0.001**	**0.6**	**0.762**	**0.759**	**80.982**

AUC = area under the curve, AIC = Akaike information criterion, CBV = cerebral blood volume, TTP = time to peak, AUC_DCE_ = area under the curve, ADC = apparent diffusion coefficient, and MK = mean kurtosis. ^1^ Apart from the combined approaches, cut-off values of the raw data are shown, not those of the univariate binary logistic regressions. Weights of the logistic regression in parentheses: ^2^ AUC_DCE_ (−0.006) and MK (−0.003); the parameter/combination with the highest AUC and lowest AIC is highlighted in thick letters.

**Table 4:** Diagnostic performance of all dynamic contrast-enhanced (DCE) MRI and diffusion-weighted imaging (DWI) parameters and the combined approaches based on the multivariant binary regression analyses in predicting oligodendroglioma (IDH1/2 mutated 1p/19q codeletion) (WHO grade 3) vs. glioblastoma IDH1/2 wildtype (WHO grade 4) and astrocytoma IDH1/2 mutated (WHO grades 3 and 4).

	Oligodendroglioma (IDH1/2 Mutated 1p/19q Codeletion) (WHO Grade 3) vs. Glioblastoma IDH1/2 Wildtype (WHO Grade 4) and Astrocytoma IDH1/2 Mutated (WHO Grades 3 and 4)					
	AUC (95% Confidence Interval)	*p* Value	Cut-Off Value ^1^	Sensitivity	Specificity	AIC
**Ktrans**	0.739 (0.598–0.880)	0.010	834.1	0.750	0.745	57.060
**Kep**	0.655 (0.471–0.838)	0.095	1665.0	0.667	0.800	60.861
**Vp**	0.642 (0.490–0.795)	0.124	1269.2	0.917	0.436	61.163
**Ve**	0.680 (0.505–0.855)	0.052	134.7	0.667	0.836	62.006
**CBV**	0.720 (0.561–0.878)	0.018	900.6	0.750	0.745	59.183
**TTP**	0.660 (0.473–0.847)	0.085	669.4	0.667	0.782	60.728
**Peak**	0.733 (0.571–0.894)	0.012	10.9	0.583	0.873	58.150
**AUC_DCE_**	0.721 (0.555–0.887)	0.017	41.4	0.583	0.836	61.297
**wash in**	0.715 (0.587–0.843)	0.020	471.6	0.750	0.727	57.818
**wash out**	0.433 (0.262–0.605)	0.472	44.0	1.000	0.127	64.886
**ADC**	0.721 (0.555–0.887)	0.054	505.4	0.583	0.836	60.645
**MK**	0.741 (0.592–0.890)	0.009	658.3	0.750	0.655	57.369
**Ktrans and MK ^2^**	**0.806 (0.700–0.912)**	**<0.001**	**0.1**	**1.000**	**0.600**	**54.839**

AUC = area under the curve, AIC = Akaike information criterion, CBV = cerebral blood volume, TTP = time to peak, AUC_DCE_ = area under the curve, ADC = apparent diffusion coefficient, and MK = mean kurtosis. ^1^ Apart from the combined approaches, cut-off values of the raw data are shown, not those of the univariate binary logistic regressions. Weights of the logistic regression in parentheses: ^2^ Ktrans (−0.005) and MK (−0.001); the parameter/combination with the highest AUC and lowest AIC is highlighted in thick letters.

## Data Availability

In order to safeguard the confidentiality of the participants, the data pertaining to this study are currently withheld from public access. The data can be shared upon request.

## Supplementary Materials

The following supporting information can be downloaded at: https://www.mdpi.com/article/10.3390/cancers16152644/s1, Figure S1: Exemplary regions of interest in biopsy sites of different patients. Figure S2: Group comparison of diffusion-weighted imaging/diffusion kurtosis imaging parameters, dynamic contrast-enhanced MRI parameters and neurite orientation dispersion and density imaging parameters between patients with high-grade gliomas and low-grade gliomas. An asterisk (*) marks significant correlation at *p* < 0.004; Figure S3: Group comparison of diffusion-weighted imaging/diffusion kurtosis imaging parameters, dynamic contrast-enhanced MRI parameters and neurite orientation dispersion and density imaging parameters between patients with IDH 1/2 mutated and IDH 1/2 wildtype gliomas. An asterisk (*) marks significant correlation at *p* < 0.004; Figure S4: Group comparison of diffusion-weighted imaging/diffusion kurtosis imaging parameters, dynamic contrast-enhanced MRI parameters and neurite orientation dispersion and density imaging parameters between patients with high-grade oligodendroglial and high-grade astrocytic gliomas. An asterisk (*) marks significant correlation at *p* < 0.004; Table S1: Diagnostic performance of all dynamic contrast-enhanced (DCE) MRI and diffusion-weighted imaging (DWI) parameters.
