# Supplementary material for: Glioma Type Prediction with Dynamic Contrast-Enhanced MR Imaging and Diffusion Kurtosis Imaging—A Standardized Multicenter Study
Source: Cancers (Basel). 2024 Jul 25;16(15):2644. doi: 10.3390/cancers16152644 (PMC11311685; doi:10.3390/cancers16152644)
Supplement: Supplementary file 1 [file cancers-16-02644-s001.zip › 4. Table S1.docx]

**Table S1.** Diagnostic performance of all dynamic contrast-enhanced (DCE) MRI and diffusion-weighted imaging (DWI) parameters

|  | **WHO grade 2 vs. WHO grade 3 adult-type gliomas** | | **IDH1/2 wild type (WHO grade 3) vs. IDH1/2 mutated adult-type gliomas (WHO grade 2 and 3)** | | **Oligodendroglioma (IDH1/2 mutated 1p/19q codeletion) (WHO grade 3) vs. and Astrocytoma IDH1/2 mutated (WHO grade 3)** | |
| --- | --- | --- | --- | --- | --- | --- |
|  | **AUC (95 % Confidence interval)** | **p value** | **AUC (95 % Confidence interval)** | **p value** | **AUC (95 % Confidence interval)** | **p value** |
| **Ktrans** | 0.750 (0.506-0.994) | 0.076 | 0.822 (0.661-0.983) | 0.024 | 0.625 (0.357-0.893) | 0.355 |
| **Kep** | 0.583 (0.323-0.844) | 0.554 | 0.533 (0.244-0.823) | 0.815 | 0.594 (0.324-0.863) | 0.487 |
| **Vp** | 0.696 (0.416-0.976) | 0.163 | 0.756 (0.458-1.000) | 0.073 | 0.677 (0.408-0.956) | 0.190 |
| **Ve** | 0.815 (0.592-1.000) | 0.025 | 0.800 (0.628-0.972) | 0.036 | 0.510 (0.232-0.789) | 0.939 |
| **CBV** | 0.774 (0.531-1.000) | 0.052 | 0.844 (0.706-0.983) | 0.016 | 0.615 (0.334-0.895) | 0.396 |
| **TTP** | 0.738 (0.505-0.971) | 0.091 | 0.548 (0.319-0.777) | 0.736 | 0.609 (0.349-0.870) | 0.418 |
| **Peak** | 0.869 (0.706-1.000) | 0.083 | 0.785 (0.615-0.956) | 0.046 | 0.615 (0.365-0.864) | 0.396 |
| **AUC_DCE_** | 0.810 (0.589-1.000) | 0.028 | 0.800 (0.633-0.967) | 0.036 | 0.599 (0.342-0.856) | 0.464 |
| **wash in** | 0.798 (0.563-1.000) | 0.035 | 0.770 (0.576-0.965) | 0.058 | 0.583 (0.300-0.867) | 0.537 |
| **wash out** | 0.589 (0.309-0.869) | 0.562 | 0.611 (0.335-0.887) | 0.436 | 0.661 (0.408-0.915) | 0.232 |
| **ADC** | 0.976 (0.917-1.000) | 0.001 | 0.948 (0.872-1.000) | 0.002 | 0.823 (0.634-1.000) | 0.017 |
| **MK** | 0.964 (0.884-1.000) | 0.001 | 0,956 (0.883-1.009) | 0.001 | 0.865 (0.706-1.000) | 0.007 |

AUC= area under the curve, CBV = cerebral blood volume, TTP == time to peak, AUC_DCE_ = area under the curve, ADC = apparent diffusion coefficient, MK = mean kurtosis.
