# Supplementary figures and images for: Glioma Type Prediction with Dynamic Contrast-Enhanced MR Imaging and Diffusion Kurtosis Imaging—A Standardized Multicenter Study
Source: Cancers (Basel). 2024 Jul 25;16(15):2644. doi: 10.3390/cancers16152644 (PMC11311685; doi:10.3390/cancers16152644)

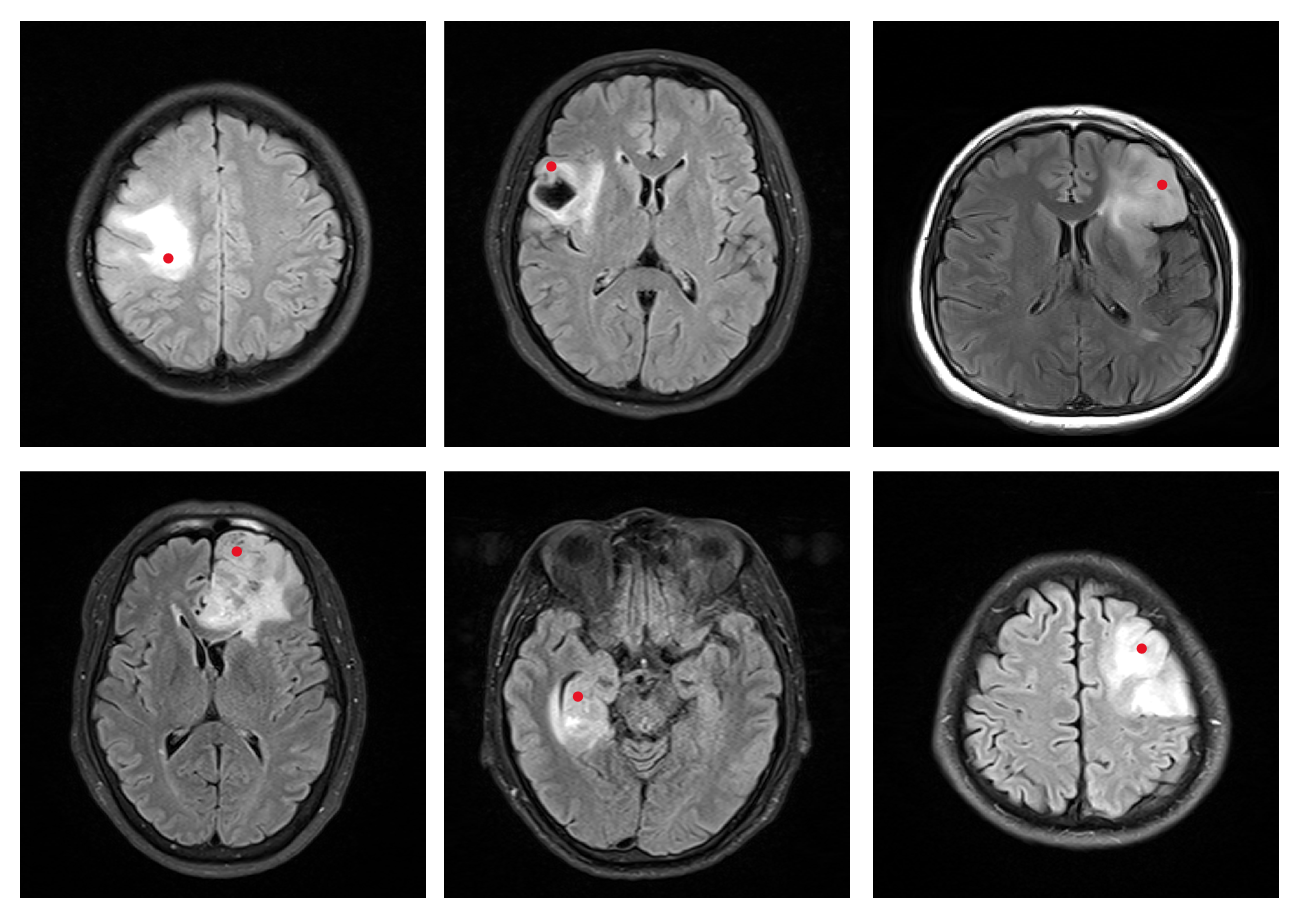

Supplement: Supplementary file 1 [file cancers-16-02644-s001.zip › 4.S1.tif]

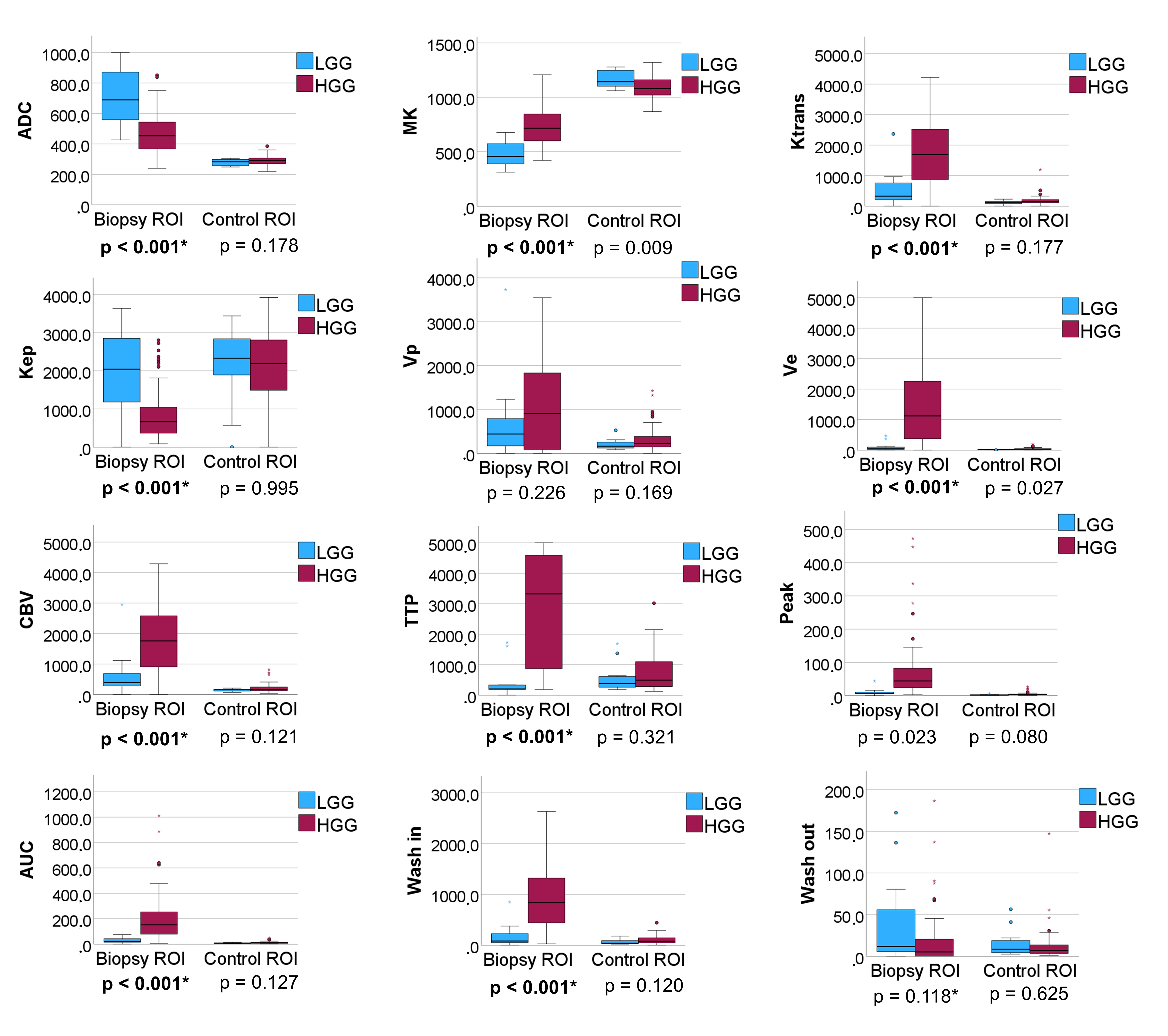

Supplement: Supplementary file 1 [file cancers-16-02644-s001.zip › 4.S2.tif]

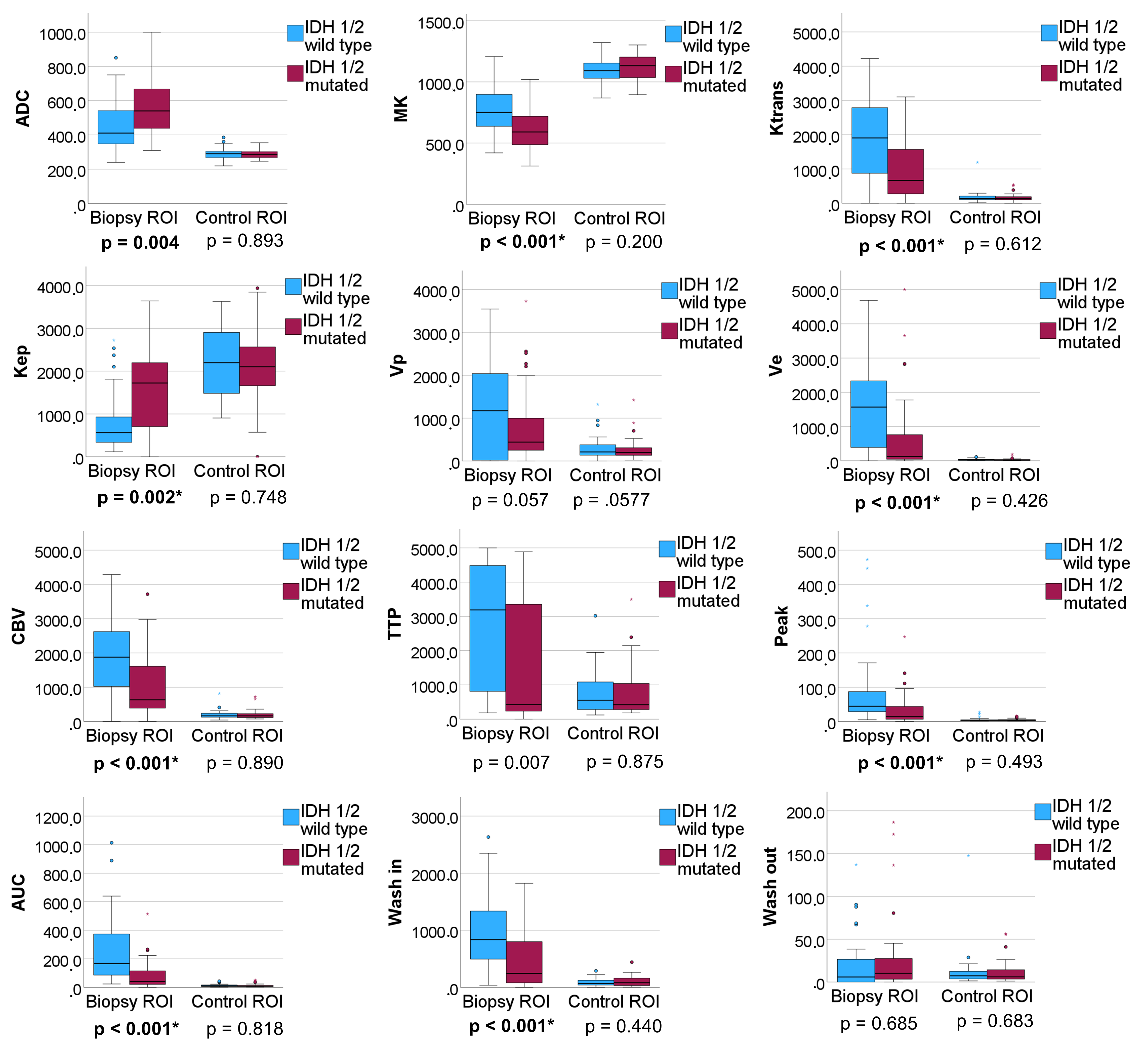

Supplement: Supplementary file 1 [file cancers-16-02644-s001.zip › 4.S3.tif]

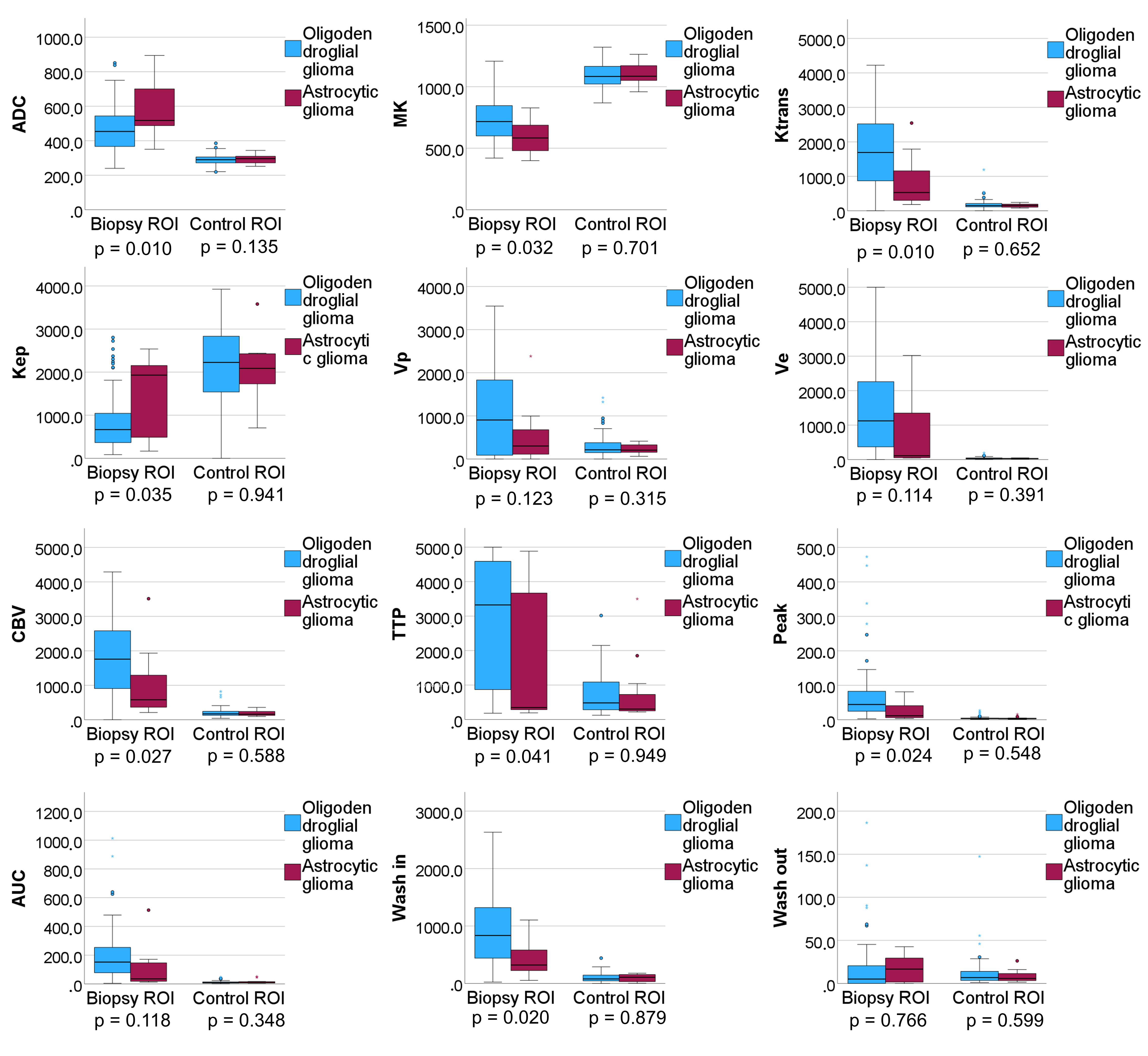

Supplement: Supplementary file 1 [file cancers-16-02644-s001.zip › 4.S4.tif]
